# The effects of crocin, insulin and their co-administration on the heart function and pathology in streptozotocin-induced diabetic rats

**Published:** 2016

**Authors:** Amir Abbas Farshid, Esmaeal Tamaddonfard, Masoumeh Moradi-Arzeloo, Navideh Mirzakhani

**Affiliations:** 1 *Division of Pathology, Department of Pathobiology, Faculty of Veterinary Medicine, Urmia University, Urmia, Iran*; 2 *Division of Physiology, Department of Basic Sciences, Faculty of Veterinary Medicine, Urmia University, Urmia, Iran*

**Keywords:** *Crocin*, *Insulin*, *Diabetic cardiomyopathy*, *Rats*

## Abstract

**Objective:**

Crocin is a saffron constituent with a potent anti-oxidant activity. The present study investigated the effects of crocin and insulin treatments (alone or in combination) on cardiac function and pathology in diabetic rats.

**Materials and Methods:**

Diabetes was induced by intraperitoneal (i.p.) injection of streptozotocin (STZ, 50 mg/kg). Thereafter, crocin (5, 10 and 20 mg/kg, i.p.), subcutaneous (s.c.) injection of insulin (4 IU/kg) and their combination were administered for eight weeks. Blood glucose level and whole heart and body weights were measured. Electrocardiography (ECG) was carried out using the lead II. Serum concentrations of lactate dehydrogenase (LDH), creatine kinase-MB isoenzyme (CK-MB), and the heart tissue malodialdehyde (MDA) and superoxide dismutase (SOD) contents were determined. The heart lesions were evaluated by light microscopy.

**Results:**

STZ decreased body weight and increased whole heart weight/body weight ratio. It also decreased heart rate, and increased RR and QT intervals and T wave amplitude. STZ increased blood glucose, serum LDH and CK-MB levels, augmented heart tissue MDA content, decreased SOD content of heart tissue, and produced hemorrhages, degeneration, interstitial edema, and fibroblastic proliferation in the heart tissue. Crocin (10 and 20 mg/kg, i.p.), insulin (4 IU/kg, s.c.) and their combination (5 mg/kg of crocin with 4 IU/kg of insulin) treatments recovered the ECG, biochemical and histopathological changes induced by STZ.

**Conclusion:**

The results showed cardioprotective effects of crocin and insulin in STZ-induced diabetic rats. The antioxidant and anti-hyperglycemic properties of crocin and insulin may be involved in their cardioprotective actions.

## Introduction

It is well known that diabetes mellitus (DM) is a major health problem (Ng et al., 2014 ▶). Worldwide, the number of diabetic patients is increasing very fast and expected to reach 439 million by 2030 (Shawm et al., 2010 ▶). Retinopathy, nephropathy, neuropathy and cardiomyopathy are the most common complications in diabetic patients (Forouhi and Wareham, 2014 ▶). Diabetic cardiomyopathy (DCM) describes diabetes-associated changes in the structure and function of the myocardium (Liu et al., 2014 ▶). Although DCM is increasingly recognized, the underlying mechanisms are still obscure. In this context, glucotoxicity due to chronic hyperglycemia, lipotoxicity resulting from hyperlipidemia, hyperinsulinemia, abnormalities in intracellular Ca^2+^ hemostasis, mitochondrial dysfunction and oxidative stress are involved in the pathogenesis of DCM (Liu et al., 2014 ▶; Letonja and Petrovic, 2014 ▶). Several therapeutic strategies such as exercise, and administration of antioxidants, anti-diabetic drugs, medicinal plants and their active constituents have been implicated in the treatment of DCM (Liu et al., 2014 ▶; Adegate et al., 2010 ▶).

Crocin is one of the major biologically active substances of *Gardenia jasminoids* fruits extract and *Crocus sativus* stigmas extract (Liu et al., 2013 ▶; Gonda et al., 2012 ▶). This phytochemical compound exerts many pharmacological effects including anti-oxidant, anti-cancer and neuroprotective activities (Zhang et al., 2013 ▶; Farshid and Tamaddonfard, 2015 ▶; Rahaiee et al., 2015 ▶; Wang et al., 2015 ▶). Although there are no reports showing the effects of crocin on DCM, some experimental studies have suggested beneficial effects of crocin in the treatment of diabetes and its complications. Crocin showed anti-hyperglycemic, antioxidant and anti-hyperlipidemic effects in STZ-induced diabetic rats (Rajaei et al., 2013 ▶; Shirali et al., 2013 ▶; Asri-Rezaei et al., 2015 ▶). In addition, renal injuries (nephropathy) in diabetic rats were attenuated by crocin (Altinoz et al., 2015 ▶). Insulin, as the major glucoregulatory hormone, was found to exert cardioprotective effects on myocardial ischemia/reperfusion injury via reducing oxidative/nitrative stress (Ji et al., 2010 ▶). It also protected the heart from the effects of STZ-induced DCM (Semaming et al., 2014 ▶). However, tight control of blood glucose partially eliminated cardiac dysfunction. In this regard, new treatment strategies including combination therapy are required for the treatment of diabetic cardiomyopathy (Zaboli et al., 2002 ▶; Kim et al., 2008 ▶; Kavak et al., 2012 ▶; Akhtar et al., 2016 ▶). 

In the present study, we investigated the effects of crocin and insulin treatments (alone or in combination) on STZ-induced cardiac pathology in diabetic rats by measuring blood glucose level, body weight, whole heart weight/body weight ratio, and serum levels of LDH, CK-MB and evaluation of heart tissue contents of MDA and SOD as well as ECG and histopathological changes of the heart.

## Materials and Methods

**Animals**

Healthy adult male Wistar rats, weighing 250–270 g were used in this study. Rats were grouped as four animals per cage and kept in a 12 hr/12 hr light-dark cycle (light on at 07:00 h) at a controlled ambient temperature (22 ± 0.5°C) with *ad libitum* access to food and water. All research and animal care procedures were approved by the Veterinary Ethics Committee of the Faculty of Veterinary Medicine of Urmia University, Urmia, Iran and were performed in accordance with the National Institutes of Health Guide for Care and Use of Laboratory Animals.

**Chemicals**

Crocin and STZ were purchased from FlukaRiedel-de Haën (Buchs SG, Schweiz) and Sigma–Aldrich Inc. (St Louis, MO, USA), respectively. Insulin was obtained from Exir Co. Pvt. Ltd. (Tehran, Iran). Crocin, STZ and insulin were dissolved in normal saline. All analytical-grade chemicals were purchased from Merck Chemical Co. (Darmstadt, Germany). 

**Treatment groups**

In the present study, 56 male Wistar rats were divided into seven groups of eight rats. Group 1 (intact group) received citrate buffer followed by normal saline. Group 2 (STZ group) received STZ (50 mg/kg) followed by normal saline. Groups 3, 4 and 5 (crocin groups) received STZ followed by 5, 10 and 20 mg/kg of crocin, respectively. Group 6 (insulin group) received STZ followed by 4 IU/kg of insulin. Group 7 (crocin + insulin group) received STZ followed by crocin (5 mg/kg) plus insulin (4 IU/kg). Five days a week injection (i.p.) of crocin and daily subcutaneous injection (s.c.) of insulin were given for eight weeks after confirmation of diabetes. The doses and administration routes of chemicals used in the present study, were chosenaccording to previous studies in which crocin (7.5-30 and 15-60 mg/kg, i.p.) and insulin (3, 4 and 5 IU/kg, s.c.) were used (Rajaei et al., 2013 ▶; Semaming et al., 2014 ▶; Tamaddonfard et al., 2013 ▶; Wayhs et al., 2013 ▶; Erken et al., 2015 ▶; Farshid et al., 2015 ▶). 

**Induction of diabetes**

Diabetes mellitus was induced in overnight-fasted rats by a single injection of freshly prepared STZ (50 mg/kg, i.p.) (Szkudelski, 2001). STZ was dissolved in sodium citrate buffer (0.1 M, pH 4.5). Hyperglycemia was confirmed by elevated glucose levels in plasma, determined 72 hr after injection of STZ, using a digital glucometer (Elegans, Germany). The animals with blood glucose concentration of >250 mg/dl were used for the study. We also determined the level of blood glucose by the same method at the end of study (day 56 after confirmation of diabetes). 

**Electrocardiography**

ECG was recorded five days before injection (i.p.) of STZ, and 24 hr after the last treatment with the above-mentioned agents using ECG apparatus (Cardio, Zimence, Germany) as described by Farshid et al. (2014). ▶ The rats were anaesthetized with ketamine (80 mg/kg, i.p.) and xylazine (8 mg/kg, i.p.). In all animals, 15 min after anesthesia, 30-guage needle electrodes were inserted under skin for the limb lead at position II. The ECG apparatus was calibrated at 1 mV/1 cm with speed of 50 mm/s. After 5min, the ECG was recorded for five seconds. Heart rate, RR and QT intervals and T wave amplitude were calculated from ECG recordings. Corrected QT interval (QTc), which is used to rectify the influence of the heart rate on QT interval, according to Bazett formula, is equal to QT interval divided by the square root of RR interval (Kmecova and Klimas, 2010 ▶), was also calculated. Some researchers have evaluated the above mentioned ECG changes in STZ-induced (Howarth et al., 2005 ▶, 2009a ▶, 2009b ▶; VanHoose et al., 2010 ▶; Lin et al., 2012 ▶; Jankyova et al., 2012 ▶). 

**Blood and tissue sampling**

At the end of the experiments (day 56 after induction of diabetes), after measuring fasting blood glucose levels and electrocardiography recordings, a 23-gauge, injection needle was inserted into the heart through 7^th^ and 8^th^intercostals muscles (Farshid et al., 2014 ▶). Blood samples were collected from the heart using non-heparin containing tubes. These tubes were centrifuged at 3500 rpm for 10 min, separated serum samples and transferred to Eppendorf tubes for biochemical analysis of LDH and CK-MB. Immediately after blood sampling, the upper abdomen was opened and the heart was removed, washed with normal saline, blotted dry on filter papers and weighted. Thereafter, one half of the heart was washed to prepare homogenates for biochemical analysis, and the remaining half was fixed in 10% formalin and used for histological studies.

**Biochemical assay**

Serum levels of LDH and CK-MB were measured spectrophotometrically (LKB Ultrasepec, Austria) using their test kits that was obtained from Man Co., Tehran, Iran and Pars Azmoon Co., Tehran, Iran, respectively. Serum levels of LDH and CK-MB level were expressed as units per liter (U/l). 

MDA levels in the heart tissue homogenates were determined by thiobarbitoric acid (TBA) method (Ohkawa et al., 1979 ▶). Heart was homogenized in 10% trichloroacetic acid (TCA) at4°C. A 0.2 ml homogenate was pipetted into a test tube, followed by the addition of 0.2 ml of 8.1% sodium dudecylsulphate, 1.5 ml of 30% acetic acid (pH 3.5) and 1.5 ml of 0.8% TBA. Tubes were boiled for 60 min at 95°C and then were cooled on ice. Then, 1ml of distilled water and 5 ml of n-butanol:pyridine (15:1 v/v) mixture were added to the tubes and centrifuged at 1500 rpm for 10 min. The absorbance of the developed color in organic layer was measured at 532 nm. MDA level is expressed as nmol/g tissue. SOD activity was determined in the supernatant using the nitroblue tetrazolium (NBT) based on the method described by Delides et al. (1976) ▶. This method employs xanthine and xanthine oxidase to generate superoxide radicals which react with 2-(4-iodophenyl)-3-(4-nitrophenole)-5-phenyltetrazolium chloride (INT) to form a red formazen dye. The SOD activity is then measured by the degree of inhibition of this reaction. One unit of SOD causes a 50% inhibition of the rate of reduction of INT under the conditions of the assay. SOD activity was expressed as U/mg protein. Protein content in the heart tissue homogenate was estimated by the method described by Lowry et al. (1951) ▶.

**Histopathology evaluation**

Fixed heart tissues were processed for paraffin embedding. For each sample, 4-5 µm thick sections were cut, stained by hematoxylin and eosin, and examined under a light microscope. Cardiac sections from eachanimal were provided. The evaluation of the heart sections was based on the severity of the pathological changes including hemorrhages, degeneration, interstitial edema and fibroblastic proliferation. The following scores were given to the severity of histopathological lesions: 0: none, 1: mild, 2:moderate and 3:severe.

**Statistical analysis**

Statistical comparisons were performed using the GraphPad Prism version 5 software (GraphPad Software, San Diego, CA, USA). Unpaired t-test was applied todo statistical analysis between intact and STZ groups. One-way ANOVA and then Tukey’s test were applied to analyze the differences among crocin and insulin (alone and combination)-treated groups and STZ group. In figures, all values were expressed as mean ± SEM. A p<0.05 was considered statistically significant.

## Results

As shown in Table 1, STZ (p<0.0001) increased blood glucose level and decreased body weight. Crocin (10 and 20 mg/kg, but not 5 mg/kg), insulin (4 IU/kg), and crocin (5 mg/kg) plus insulin (4 IU/kg) significantly (p<0.001) improved the STZ-induced changes in blood glucose level and body weight. The improving effects of combination treatment (crocin plus insulin) on the above-mentioned parameters were more significant (p<0.001) than those obtained by treatment with crocin alone.

As shown in Table 2, STZ non-significantly decreased whole heart weight. Alone and combined treatments with crocin and insulin produced no significant effects on whole heart weight. STZ significantly (p<0.0001) increased whole heart weight/body weight ratio. Crocin (10 and 20 mg/kg, but not 5 mg/kg), insulin (4 IU/kg), and crocin (5 mg/kg) plus insulin (4 IU/kg) significantly (p<0.001) improved STZ-induced changes in whole heart weight/body weight ratio. The improving effect of combination therapy (crocin plus insulin) was more significant (p<0.001) than crocin alone. 

**Table 1:** Effects of crocin and insulin on blood glucose level and body weight in streptozotocin-induced diabetic rats.

**Groups **	**Blood glucose (mg/dl) **	** Body weight (g) **
**Citrate buffer +Normal saline**	110 ± 7.8	373 ± 16.5
**STZ+ Normal saline**	373 ± 16.5^a^	164 ± 9.3^a^
**STZ + Crocin(5 mg/kg)**	385 ± 20.8	191 ± 10.5
**STZ + Crocin (10 mg/kg)**	344 ± 18.5^b^	242 ± 15.3^b^
**STZ + Crocin (20 mg/kg)**	296 ± 15.8^c^	289 ± 16.2^c^
**STZ + Insulin (4 IU/kg)**	256 ± 13.4^c^	309 ± 14.8^c^
**STZ + Crocin (5 mg/kg)+Insulin (4IU/kg) **	236 ± 13.8^c^^d^	318 ± 16.7^c^^d^

Values are given as mean ± SEM (n = 8).

a p<0.0001 as compared to citrate buffer + normal saline.

b p<0.01,

c p<0.001 as compared to STZ + normal saline.

d p<0.001 as compared to STZ + crocin (5 mg/kg) alone. STZ: streptozotocin.

**Table 2:** Effects of crocin and insulin on whole heart weight and whole heart weight/body weight ratio in streptozotocin-induced diabetic rats

**Groups **	**Whole heart weight (mg) **	**Whole heart weight (mg) ** **/Body weight (g) **
**Citrate buffer +Normal saline**	853 ± 15.9	2.32 ± 0.12
**STZ + Normal saline**	827 ± 16.2	5.16 ± 0.36^a^
**STZ + Crocin (5 mg/kg)**	830 ± 14.3	4.45 ± 0.31
**STZ + Crocin (10 mg/kg)**	836 ± 14.5	3.55 ± 0.21^b^
**STZ + Crocin (20 mg/kg)**	841 ± 16.7	2.99 ± 0.19^b^
**STZ + Insulin (4 IU/kg)**	826 ± 14.2	309 ± 14.8^c^
**STZ + Crocin (5 mg/kg) + Insulin (4 IU/kg) **	839 ± 15.3	2.69 ± 0.15^b^^c^

Values are given as mean ± SEM (n = 8).

a p<0.0001 as compared to citrate buffer + normal saline.

b p<0.001 as compared to STZ + normal saline.

c p<0.001 as compared to STZ + crocin (5 mg/kg) alone. STZ: streptozotocin.

**Figure 1:**
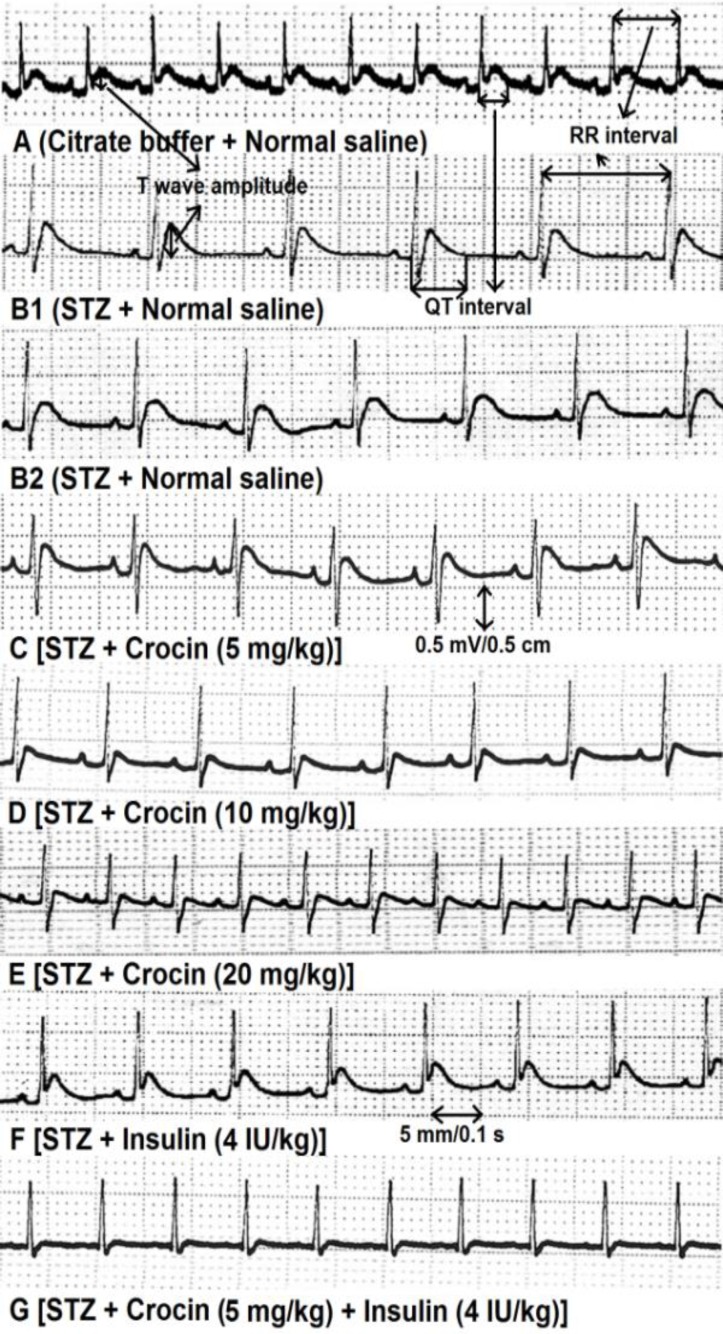
ECG recordings from control, STZ, crocin and insulin-treated rats. Figure 1A shows normal ECG recordings. STZ (Figure 1B1 and 1B2) produced cleared changes in heart rate, RR and QT intervals and T wave amplitude. Crocin (Figure 1C, 1D and 1E), insulin (Figure 1F) and crocin plus insulin (Figure 1G) reversed the ECG changes induced by STZ. RR interval shows the distance between the peaks of two consecutive R waves. QT interval shows the distance between the beginning of Q wave and the last of T wave. STZ: streptozotocine; speed: 5 mm / 0.1 s (50 mm / 1 s); amplitude: 0.5 mV/ 0.5 cm (1 mV / 1 cm

Figures 1 and Table 3 show the effects of normal saline, STZ, crocin, insulin and crocin plus insulin treatments on ECG recordings. Normal saline-treated rats showed normal ECG pattern (Figure 1A and Table 3). STZ significantly (p<0.0001) decreased heart rate and increased RR and QT intervals as well as T wave amplitude (Figure 1B1 and1B2 and Table 3). Crocin (5 mg/kg) (Figure 1C, Table 3) had no significant effects on ECG recording changes, whereas at doses of 10 mg/kg (Figure 1D and Table 3) and 20 mg/kg (Figure 1E and Table 3), it significantly (p<0.001) improved the effects of STZ on ECG recording changes. Insulin at a dose of 4 IU/kg (Figure 1F and Table 3) significantly (p<0.001) improved heart rate, RR interval and T wave amplitude changes induced by STZ but had no effects on QT and QTc intervals. 

Treatment with crocin (5 mg/kg) plus insulin (4 IU/kg) produced more significant (p<0.01) recovering effect on ECG recordings as compared to each chemical used alone (Figure 1G and Table 3).

**Figure 2:**
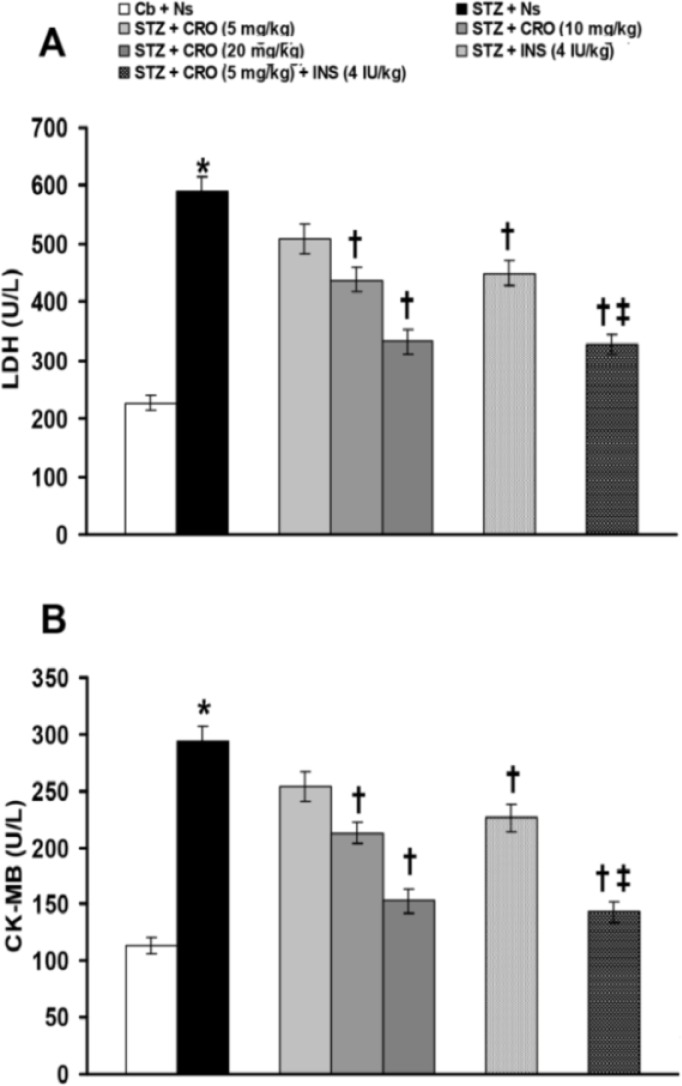
Effects of alone and combined treatments with crocin and insulin on serum level changes of LDH (A) and CK-MB (B) induced by STZ. Data are presented as mean ± SEM (n = 8). *p<0.0001 as compared to citrate buffer + normal saline group.^†^p<0.001 as compared to STZ + normal saline group. ‡p<0.001 as compared with STZ + crocin (5 mg/kg) and insulin (4 IU/kg) alone. Cb: citrate buffer; Ns: normal saline; STZ: streptozotocin;CRO: crocin;INS: insulin;LDH: lactate dehydrogenase;CK-MB: creatin kinase MB isoenzyme

Figure 2 shows the effects of crocin and insulin treatments (alone or in combination) on STZ-induced changes of serum LDH and CK-MB activities. Serum activities of LDH (Figure 2A) and CK-MB (Figure 2B) were significantly (p<0.0001) increased by STZ. Serum activity changes of LDH (Figure 2A) and CK-MB (Figure 2B) were significantly (p<0.001) restored by 10 and 20 mg/kg of crocin and 4 IU/kg of insulin. Treatment with crocin (5 mg/kg) plus insulin (4 IU/kg) produced more significant (p<0.001) restoring effects than those of their alone treatment on serum activities of LDH (Figure 2A) and CK-MB (Figure 2B) induced by STZ.

**Figure 3:**
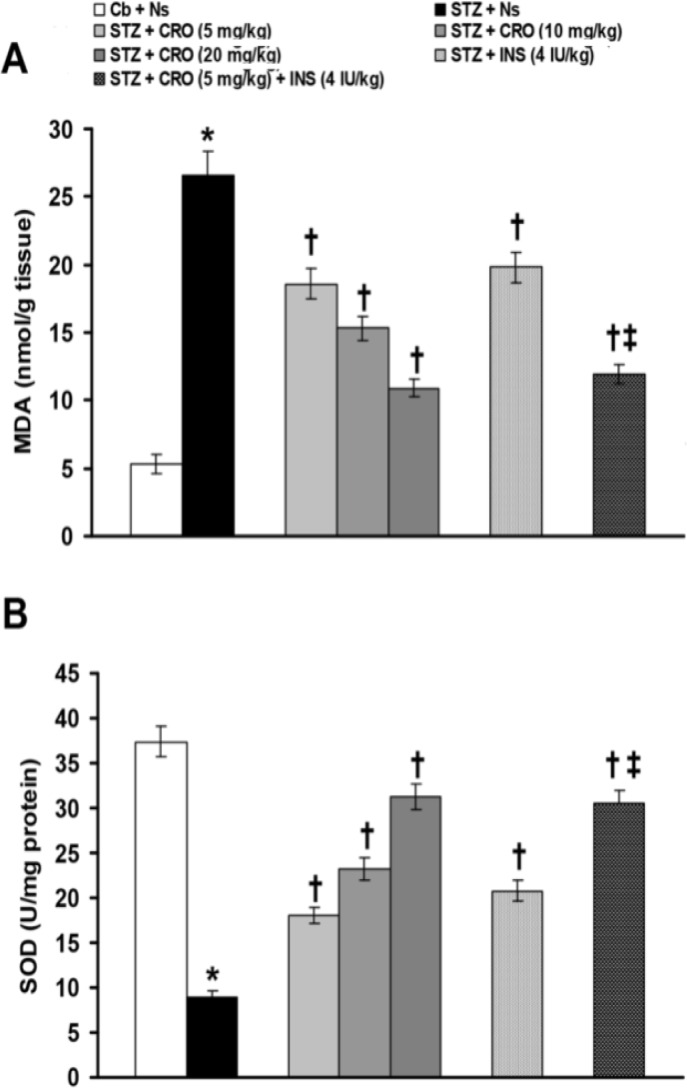
Effects of alone and combined treatments with crocin and insulin on changes of heart content of MDA (A) and SOD (B) induced by STZ. Data are presented as mean ± SEM (n = 8). *p<0.0001 as compared to citrate buffer + normal saline group.^†^p<0.001 as compared to STZ + normal saline group.^‡^p<0.001 as compared to STZ + crocin (5 mg/kg) and insulin (4 IU/kg) alone. Cb: citrate buffer;Ns: normal saline;STZ: streptozotocin;CRO: crocin;INS: insulin;MDA: malodialdehyde;SOD: superoxide dismutase

Figure 3 shows the effects of alone and combined treatments with crocin and insulin on STZ-induced changes of MDA level and SOD activityin the heart tissue. STZ significantly (p<0.0001) increased MDA level (Figure 3A) and decreased SOD activity (Figure 3B) in heart tissue. Crocin (5, 10 and 20 mg/kg), insulin (4 IU/kg) significantly (p<0.001) recovered heart tissue MDA level (Figure 3A) and SOD activity (Figure 3B) changes induced by STZ. 

Combined treatment with crocin and insulin produced more significant (p<0.001) recovering effect on heart tissue MDA level (Figure 3A) and SOD activity (Figure 3B) changes induced by STZ, as compared to each chemical alone.

**Figure 4:**
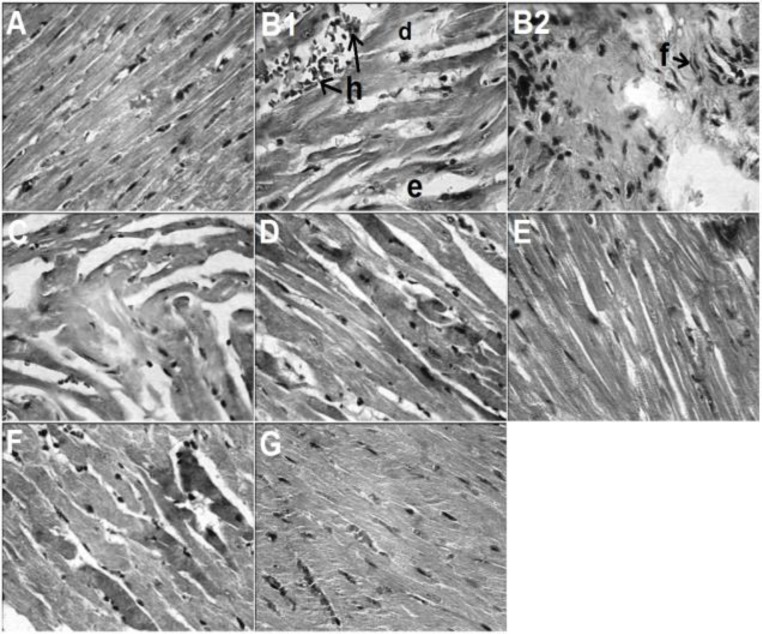
Effects of alone and combined treatments with crocin and insulin on STZ-induced histopathological alternations in cardiac tissues. (A): Intact animals received normal saline. Normal architecture of the heart is seen. (B1 and B2): Animals received STZ. Hemorrhages (h), degeneration (d), interstitial edema (e) and fibroblastic proliferation (f) are seen. (C, D and E): Animals received STZ + crocin (5 mg/kg), STZ + crocin (10 mg/kg), STZ + crocin (20 mg/kg) and insulin (4 IU/kg), respectively. Partial to complete reduction of histopatological changes are seen. (F): Aniamls received STZ + crocin (5 mg/kg) plus STZ + insulin (4 IU/kg). Complete reduction of histopathological changes is evident (H&E × 400

Figure 4 and Table 4 show the effects of crocin and insulin treatments (alone or in combination) on the heart tissue histopathological changes induced by STZ. Citrate buffer plus normal saline-treated group showed normal histology of the heart (Figure 4A and Table 4). STZ produced hemorrhages, degeneration, interstitial edema and fibroblastic proliferation in the heart tissue (Figure 4B1 and 4B2 and Table 4).

Histopathological changes induced by STZ were not recovered by crocin (5 mg/kg) (Figure 4C and Table 4). STZ-induced histopathological changes in the heart tissue were significantly (p<0.001) improved by 10 mg/kg (Figure 4D and Table 4) and 20 mg/kg (Figure 4E and Table 4) of crocin, 4 IU/kg of insulin (Figure 4F and Table 4) and 5 mg/kg of crocin plus 4 IU/kg of insulin (Figure 4G and Table 4).

**Table 3:** The effects of crocin and insulin on heart rate, RR, QT and QTc intervals, and T wave amplitude in streptozotocin-induced diabetic cardiomyopathy in rats

**Groups **	**Heart rate(bpm)**	**RRinterval(s) **	**QTinterval(s) **	**QTcinterval(s) **	**T waveamplitude**
**Citrate buffer +Normal saline**	383 ± 15.4	0.159 ± 0.006	0.066 ± 0.004	0.165 ± 0.007	0.113 ± 0.007
**STZ + Normal saline**	229 ± 11.6^a^	0.267 ± 0.014^a^	0.123 ± .007^a^	0.241 ± 0.011	0.289 ± 0.015^a^
**STZ + Crocin (5 mg/kg)**	264 ± 10.2	0.229 ± 0.008	0.116 ± 0.005	0.239 ± 0.014	0.266 ± 0.014
**STZ + Crocin (10 mg/kg)**	290 ± 12 b	0.211 ± 0.009^b^	0.085 ± .004^b^	0.187 ± 0.011^b^	0.194 ± 0.013^b^
**STZ + Crocin (20 mg/kg)**	360 ± 14.8^b^	0.174 ± 0.007^b^	0.078 ± .004^b^	0.174 ± 0.011^b^	0.175 ± 0.015^b^
**STZ + Insulin (4 IU/kg)**	296 ± 12.4^b^	0.205 ± 0.008^b^	0.104 ± 0.004	0.229 ± 0.014	0.224 ± 0.016^b^
**STZ + Crocin (5 mg/kg) + Insulin (4 IU/kg) **	354 ± 12.9^b^^c^	0.171 ± 0.006^b^^c^	0.071 ± .005^b^^c^	0.173 ± 0.012^b^^c^	0.128 ± 0.006^b^^c^

Values are given as mean ± SEM (n = 8).

a p<0.0001 as compared to citrate buffer + normal saline group.

b p<0.001 as compared to STZ + normal saline group.

c p<0.01 as compared to STZ + crocin (5 mg/kg) and STZ + insulin (4 IU/kg) groups. STZ: streptozotocin.

**Table 4:** Effects of crocin and insulin on hemorrhages, degeneration, interstitial edema and fibroblastic proliferation severities in streptozotocin-induced diabetic rats

**Groups **	**Hemorrhages**	**Degeneration **	**Interstitial edema **	**Fibroblastic prolifration**
**Citrate buffer +Normal saline**	0.00 ± 0.00	0.00 ± 0.00	0.00 ± 0.00	0.00 ± 0.00
**STZ + Normal saline**	2.75 ± 0.16^a^	2.75 ± 0.25^a^	2.63 ± 0.18^a^	2.50 ± 0.19^a^
**STZ + Crocin (5 mg/kg)**	2.50 ± 0.19	2.38 ± 0.18	2.13 ± 0.29	2.00 ± 0.27
**STZ + Crocin (10 mg/kg)**	1.75 ± 0.16^b^	1.63 ± 0.16^b^	1.50 ± 0.19^b^	1.38 ± 0.18^b^
**STZ + Crocin (20 mg/kg)**	1.50 ± 0.19^b^	1.38 ± 0.18^b^	1.13 ± 0.13^b^	0.88 ± 0.23^b^
**STZ + Insulin (4 IU/kg)**	1.63 ± 0.18^b^	1.50 ± 0.17^b^	1.75 ± 0.19^b^	1.25 ± 0.16^b^
**STZ + Crocin (5 mg/kg) + Insulin (4 IU/kg) **	0.75 ± 0.16^b^	0.75 ± 0.18^b^	0.63 ± 0.18^b^	0.50 ± 0.19^b^

Values are given as mean ± SEM (n = 8).

a p<0.0001 as compared to citrate buffer + normal saline group.

b p<0.001 as compared to STZ + normal saline group. STZ: streptozotocin.

## Discussion

In the present study, STZ increased blood level of glucose (hyperglycemia) and decreased body weight (body weight loss). STZ enters the B cell viaa glucose transporter (GLUT_2_) and causes degeneration of pancreatic B cells leading to hypoinsulinemia and subsequent hyperglycemia (Szkudelski, 2001 ▶). Body weight loss induced by STZ may be associated with the inability to metabolize carbohydrates, which shifts fuel sources to fatty acids and proteins as energy sources. Therefore, wasting of protein and fatty acid stores induced by insulin deficiency might lead to reduction of body weight (Warne et al., 2005 ▶). STZ increased whole heart weight/body weight ratio. The whole heart weight/body weight ratio, which was considered as an index of cardiac hypertrophy, was increased because of the reduced body weight in diabetic rats. Our findings on the above-mentioned parameter changes induced by STZ are in agreement with other studies (Akhtar et al., 2016 ▶; Kuo et al., 2009 ▶; Wu et al., 2014 ▶; Zheng et al., 2015 ▶; Gao et al., 2016 ▶). Our results showed that STZ decreased heart rate, increased RR, QT and QTc intervals and T wave amplitude. The ECG recordings provide reliable parameters for assessing STZ-induced DCM (Simova et al., 2015 ▶). Heart rate reduction and prolongation of QT interval have been reported in STZ-induced diabetic rats (Howarth et al., 2005 ▶, 2009a ▶. 2009b ▶). The QT interval provides a measure of the electrical events associated with depolarization and repolarization of the heart ventricles (Postema and Wilde, 2014 ▶). QTc is a crucial and critical factor in the assessment of repolarization changes considering safety of drugs and cardiac disorders (Kmecova and Klimas, 2010 ▶). In the present study, STZ increased serum activities of LDH and CK-MB and heart tissue level of MDA and decreased heart tissue activity of SOD. Despite the fact that LDH is not specific for myocardial damage, its measurement along with creatine phosphokinase (CPK) and CK-MB may be a more reliable indicator of myocardial damage (Farshid et al., 2014 ▶; Futterman and Lemberg, 1997 ▶). Most studies have reported increased serum activities of LDH and CK-MB in STZ-induced DCM (Akhtar et al., 2016 ▶; Wang et al., 2012 ▶; Wang et al., 2013 ▶). Increased oxidative stress and altered antioxidant pool have been found in both clinical and experimental type 1 diabetes (Khullar et al., 2010 ▶; Stadler, 2012 ▶). This was in conjunction with depletion of superoxide scavenger SOD and increase in lipid peroxidation product MDA. In this study, parallel to above-mentioned ECG and biochemical changes, the microscopic findings verified myocardial injuries including hemorrhages, interstitial edema, fibroblastic proliferation and degeneration. It is widely accepted that diabetic heart is associated with left ventricular diastolic dysfunction, cardiomyocyte hypertrophy, myocardial interstitial fibrosis and appotosis (Letonja and Petrovic, 2014 ▶). In this context, Al-Rasheed et al., (2013) ▶ reported that i.p. injection of STZ (55 mg/kg) produced cardiac pathological changes including cellular infiltration, fibrosis and degeneration. 

The results of the present study showed that crocin produced improving effects on blood glucose, body weight, whole heart weight, whole heart weight/body weight ratio, ECG changes, serum LDH and CK-MB activities, heart tissue levels of MDA and SOD and histological outcomes of cardiomyopathy induced by STZ. Although, there are no reports showing the effects of crocin on STZ-induced DCM, in other experimental cardiotoxicity, crocin produced improving effects. Crocin pretreatment produced protective effects by reducing creatin phosphokinase (CPK) activity, restoring the redox status and suppressing apoptosis in patulin-induced cardiotoxicityin mice (Goyal et al., 2010 ▶). In addition, crocin produced protective effect by restoring CK-MB activity and MDA level in the heart and improving histopathological changes including necrosis of cardiac muscle cells, hemorrhages, hypertrophy and inflammatory cells infiltration in diazinon-induced cardiotoxicity (Boussabbeh et al., 2015 ▶). Moreover, crocin improved LDH, CPK and CK-MB activities of coronary effluent, and cardiac tissue oxidative stress biomarker (MDA) as well as antioxidant enzymes (catalase and SOD) and total antioxidant capacity in a rat model of cardiac ischemia-reperfusion injury (Dianat et al., 2014a ▶). In isoproterenol-induced myocardial infarction, crocin recovered myocardial CK-MB activity and reduced the increased level of MDA in the heart. It also improved heart tissue changes such as leukocyte infiltration, edema and myocardial necrosis (Razavi et al., 2013 ▶). Moreover, in an ischemia-reperfusion model of isolated heart, crocin recovered ST segment elevation, reduced infarct size and improved cardiac dysfunction (Dianat et al., 2014b ▶). In a rat heart ischemia/reperfusion model, crocin produced a cardioprotective effect via both regulation of nitric oxide production and improving mechanical function (Esmaeilizadeh et al., 2015 ▶). In addition to improving metabolic changes such as hyperglycemia, hyperlipidemia and plasma oxidative stress markers, crocin produces recovering effects on diabetic complications such as neuropathy and nephropathy (Rajaei et al., 2013 ▶; Altinoz et al., 2015 ▶; Tamaddonfard et al., 2013 ▶). 

In the present study, insulin produced improving effects on blood glucose level, body weight, whole heart weight/body weight ratio, ECG, serum LDH and CK-MB activities, heart tissue level of MDA and activity of SOD and histopathological changes induced by STZ. These results indicated that insulin improved metabolic features (hyperglycemia and body weight loss) and restored STZ-induced diabetic complications such as DCM. Subcutaneous injection of insulin (4 IU/kg) produced improving effects on body weight loss, hyperglycemia, apoptosis and cardiac hypertrophy induced by intravenous injection of 65 mg/kg of STZ (Kuo et al., 2009 ▶). In addition, administration of insulin (4 IU/kg, s.c.) decreased the increased level of cardiac tissue MDA and restored cardiac function in STZ (50 mg/kg)-induced diabetic rats (Semaming et al., 2014 ▶). However, our study results showed improving effects of insulin on some ECG parameters such as RR interval and T wave amplitude, biochemical and histopathological changes induced by STZ. This indicates a partial cardioprotective effect of insulin in STZ-induced diabetic rats. In this context, evaluation of the heart function by echocardiography and histology in STZ-induced diabetic rats revealed a partial cardioprotective effect of insulin (Kim et al., 2008 ▶). In addition, insulin failed to improve RR, QT and QTc intervals in alloxan-induced diabetic dogs (Lengyel et al., 2007 ▶). 

In comparison with crocin alone, insulin treatment alone provided better control of metabolic changes (hyperglycemia and body weight loss) induced by STZ, but partially improved STZ-induced biochemical and histopathological changes when compared with crocin (20 mg/kg). In this context, our findings have also demonstrated that treatment with crocin plus insulin has further benefits to normalization of metabolic and cardiac and especially serum and heart tissue biochemical changes induced by STZ. There are no reports showing beneficial effect of treatment with crocin plus insulin on STZ-induced DCM. Combination treatments with insulin and other chemical compounds produce beneficial effects on STZ-induced diabetes and DCM. It has been reported that a combination of insulin and vitamin A provided more benefits than use of either agent alone in the treatment of general characteristics of as well as diabetes-induced cardiac injury (Zobali et al., 2002 ▶). In addition, Kim et al. (2008) ▶ reported that combined treatment with smooth muscle cell transplantation and insulin produced better functional results in STZ-induced DCM. In STZ (25 mg/kg, for 3 consecutive days)-induced DCM, a combination of insulin and levosimendan produced better recovering effects on hemodynamic, cardiac enzymes and myofibril damage changes (Akhtar et al., 2016 ▶). A more effective cardioprotective effect was found following treatment with a combination of crocin and vitamin E in rats (Esmaeilizadeh et al., 2015 ▶). However, it has been found that effective doses of crocin (30 mg/kg) and safranal (1 mg/kg) enhanced neuroprotective effect of insulin in diabetic neuropathy (Farshid and Tamaddonfard, 2015 ▶). The insulinomimetic effect of crocin may be associated with its improving effect on serum level of insulin in STZ-induced diabetic rats (Tamaddonfard et al., 2013 ▶). 

In conclusion, the results of the present study showed that besides to hyperglycemia and body weight loss, STZ produced DCM by changing ECG pattern, serum biomarkers of cardiac injury, heart tissue level of MDA and SOD activity as well as cardiac tissue histopathology. Treatment with crocin alone or insulin alone produced improving effect on metabolic and heart tissue outcomes induced by STZ. However, when the normalizing effect of combination therapy and the effects of each agent alone are considered, the use of these agents in combination appears to have potential advantages especially in the reduction of oxidative stress in improving DCM.

## References

[B1] Adegate E, Kalasz H, Veress G, Teke K (2010). Medicinal chemistry of drugs used in diabetic cardiomyopathy. Curr Med Chem.

[B2] Akhtar MS, Pillai KK, Hassan Q, Ansari SH, Ali J, Akhtar M (2016). Levosimendan suppresses oxidative injury, apoptotic signaling and mitochondrial degeneration in streptozotocin-induced diabetic cardiomyopathy. Clin Exp Hypertens.

[B3] Al-Rasheed NM, Al-Rasheed NM, Attia HA, Hasan IH, Al-Amin M, Al-Ajmin H, Mohamad RA (2013). Adverse cardiac responses to alpha-lipoic acid in a rat-diabetic model: possible mechanisms?. J Physiol Biochem.

[B4] Altinoz E, Oner Z, Elbe H, Ciqremis Y, Turkoz Y (2015). Protective effects of saffron (its active constituent, crocin) on nephropathy in stereptozotocin-induced diabetic rats. Human ExpToxicol.

[B5] Asri-Rezaei S, Tamaddonfard E, Ghasemsoltani-Momtaz B, Erfanparast A, Gholamalipour S (2015). Effects of crocin and zinc chloride on blood levels of zinc and metabolic and oxidative parameters in streptozotocin-induced diabetic rats. Avicenna J Phythomed.

[B6] Boussabbeh M, Ben Salem I, Neffati F, Najjar MF, Bacha H, Abid-Essefi S (2015). Crocin prevents patulin-induced acute toxicity in cardiac tissues via the regulation of oxidative damage and apoptosis. J Biochem Mol Toxicol.

[B7] Delides A, Spooner RJ, Goldberg DM, Neal FE (1976). An optimized semi-automatic rate method for serum glutathione reductase activity and its application to patients with malignant disease. J Clin Pathol.

[B8] Dianat M, Esmaeilizadeh M, Badavi M, Samarbafzadeh AR, Naghizadeh B (2014a). Protective effects of crocin on ischemia-reperfusion induced oxidative stress in comparison with vitamin E in isolated rat hearts. Jundishapur J Nat Pharm Prod.

[B9] Dianat M, Esmaeilizadeh M, Badavi M, Samarbafzadeh AR, Naghizadeh B (2014b). Protective effects of crocin on hemodynamic parameters and infarct size in comparison with vitamin E after ischemia reperfusion in isolated rat hearts. Planta Med.

[B10] Erken HA, Genc O, Erken G, Ayada C, Gundagdu G, Dogan H (2015). Ozone partially prevents diabetic neuropathy in rats. Exp Clin Endocrinol Diabetes.

[B11] Esmaeilizadeh M, Dianat M, Badavi M, Samarbafzadeh A, Naghizadeh B (2015). Effect of crocin on nitric oxide synthase expression in post-ischemic isolated rat heart. Avicenna J Phytomed.

[B12] Farshid AA, Tamaddonfard E (2015). Histopathological and behavioral evaluations of the effects of crocin, safranal and insulin on diabetic peripheral neuropathy in rats. Avicenna J Phytomed.

[B13] Farshid AA, Tamaddonfard E, Simaee N, Mansouri S, Najafi S, Asri-Rezaee S, Alavi H (2014). Effects of histidine and N-acetylcysteine on doxorubicin-induced cardiomyopathy in rats. Cardiovasc Toxicol.

[B14] Forouhi NG, Wareham NJ (2014). Epidemiology of diabetes. Medicine (Abingdon).

[B15] Futterman LG, Lemberg L (1997). SGOT, LDH, HBD, CPK, CK-MB, MB1MB2, cTnT, cTnC, cTnT. Am J Crit Care.

[B16] Gao Y, Kang L, Li C, Wang X, Sun C, Li Q, Liu R, Wang J (2016). Resveratrol ameliorates diabetes-induced cardiac dysfunction through AT1R-EEK/p38 MAPK signaling pathway. Cardiovasc Toxicol.

[B17] Gonda S, Parizsa P, Suranyi G, Cyemant G, &Vasas G (2012). Quantification of main bioactive metabolites from saffron (Crocus sativus) stigmas by a micellar electrokinetic chromatographic (MEKC) method. J Pharm Biomed Anal.

[B18] Goyal SN, Arora S, Sharma AK, Joshi S, Ray R, Bhatia J, Kumari S, Arya DS (2010). Protective effect of crocin of Crocus sativus on hemodynamics, biochemical, histological and ultrastrutural alternations in isopeoteronol-induced cardiotoxicity in rats. Phytomedicine.

[B19] Howarth FC, Jacobson M, Shafiullah M, Adeghate E (2005). Long-term effects of streptozotocin-induced diabetes on the electrocardiogram, physical activity and body temperature in rats. Exp Physiol.

[B20] Howarth FC, Adeghate E, Jacobson M (2009a). Heart rate and QT interval in streptozotocin-induced diabetic rats. J Med Sci.

[B21] Howarth FC, Jacobson M, Qureshi MA, Shafiullah M, Hameed RS, Zilahi E, Al Haj A, Nowotny N, Adeghate E (2009b). Altered gene expression may underlie prolonged duration of the QT interval and ventricular action potential in streptozotocin-induced diabetic rat heart. Mol Cell Biochem.

[B22] Jankyova S, Kmecova J, Cernecka H, Mesarosova L, Musil P, Brnoliakova Z, Kyselovic J, Babal P, Klimas J (2012). Glucose and blood pressure lowering effects of Pyenogenol are inefficient to prevent prolongation of QT interval in experimental diabetic cardiomyopathy. Pathol Res Pract.

[B23] Ji L, Fu F, Zhang L, Liu W, Cai X, Zhang L, Zheng Q, zhang H, Gao F (2010). Insulin attenuates myocardial ischemia/reperfusion injury via reducing oxidative/nitrative stress. Am J Physiol Endocrinol and Metab.

[B24] Kavak S, Ayaz L, Emre M (2012). Effects of rosiglitazone with insulin combination therapy on oxidative stress and lipid profile in left ventricular muscles of diabetic rats. Exp Diabetes Res.

[B25] Khullar M, Al-Shudiefat AA, Ludke A, Binepal G, Singal PK (2010). Oxidative stress: a key contributor to diabetic cardiomyopathy. Can J Physiol Pharmacol.

[B26] Kim BO, Verma S, Weisel RD, Fazel S, Jia ZQ, Mizuno T, Li PK (2008). Preservation of heart function in diabetic rats by combined effects of muscle cell implantation and insulin therapy. Eur J Heart Fail.

[B27] Kmecova J, Klimas J (2010). Heart rate correction of the QT duration in rats. Eur J Pharmacol.

[B28] Kuo WW, Chung LC, Liu CT, Wu SP, Kuo CH, Tsai FJ, Lu MC, Huang CY, Lee SD (2009). Effects of insulin replacement on cardiac apoptotic and survival pathways in streptozotocin-induced diabetic rats. Cell Biochem Funct.

[B29] Letonja M, Petrovic DP (2014). Is diabetic cardiomyopathy a specific entity?. World J Cardiol.

[B30] Lengyel C, Virag L, Biro T, Jost N, Magyar J, Biliczki P, Kocsis E, Skoumal R, Nanasi PP, Toth M, Kecskemeti V, Papp JG, Varro A (2007). Diabetes mellitus attenuates the repolarization reserve in mammalian heart. Cardiovasc Res.

[B31] Lin YC, Huang J, Kan H, Castranova V, Frisbee JC, Yu HG (2012). Defective calcium inactivation causes long QT in obsess insulin-resistant rat. Am J Physiol Heart CircPhysiol.

[B32] Liu H, Chen YF, Li F, Zhang HY (2013). Fructus Gardenia (Gardenia jasminoides J Ellis) phytochemistry, pharmacology of cardiovascular and safety with the perspective of new drugs development. J Asian Nat Prod Res.

[B33] Liu Q, Wang S, Cai L (2014). Diabetic cardiomyopathy and its mechanisms: role of oxidative stress and damage. J Diabetes Invest.

[B34] Lowry OH, Rossenbrough NJ, Farr AL, Randall KJ (1951). Protein measurement with the Folin phenol reagent. J Biol Chem.

[B35] Ng CS, Lee JV, Toh MP, Ko Y (2014). Cost-of-illness studies of diabetes mellitus: a systematic review. Diabetes Res Clin Pract.

[B36] Ohkawa H, Ohishi N, Yagi K (1979). Assay of lipid peroxidase in normal tissue by thiobarbituric acid reaction. Anal Biochem.

[B37] Postema PG, Wilde AA (2014). The measurement of the QT interval. Curr Cardiol Rev.

[B38] Rahaiee S, Moini S, Hashemi M, Shojaosadati SA (2015). Evaluation of antioxidant activities of bioactive compounds and various extracts obtained from saffron (Crocus sativus L): a review. J Food Sci Technol.

[B39] Rajaei Z, Hadjizadeh MA, Namati H, Hosseini M, Ahmadi M, Shafiee S (2013). Antihyperglycemic and antioxidant activity of crocininstreptozotocin-induced diabetic rats. J Med Food.

[B40] Razavi BM, Hosseinzadeh H, Movassaghi AR, Imenshahidi M, Abnous K (2013). Protective effect of crocin on diazinon induced cardiotoxicity in rats in subchronic exposure. Chem Biol Interact.

[B41] Semaming Y, Kumfu S, Pannangpetch P, Chattipakorn SC, Chattipakorn N (2014). Protocatechuic acid exerts a cardioprotective effect in type 1 diabetic rats. J Endocrinol.

[B42] Shawm JE, Sicree RA, Zimmet PZ (2010). Global estimates of the prevalence of diabetes for 2010 and 2030. Diabetes Res Clin Pract.

[B43] Shirali S, Zahra Bathaie S, Nakhjavani M (2013). Effect of crocin on the insulin resistance and lipid profile of streptozotocin-induced diabetic rats. Phytother Res.

[B44] Simova I, Christov I, Bortolan G (2015). A review on electrocardiographic changes in diabetic patients. Curr Diabetes Rev.

[B45] Stadler K (2012). Oxidative stress in diabetes. Adv Exp Med Biol.

[B46] Szkudelski T (2001). The mechanisms of alloxan and streptozotocin action in B cells of the rat pancreas. Physiol Res.

[B47] Tamaddonfard E, Farshid AA, Asri-Rezaee S, Javadi S, Khosravi V, Rahman B, Mirfakhraee Z (2013). Crocin improved learning and memory impairments in streptozotocin-induced diabetic rats. Iran J Basic Med Sci.

[B48] VanHoose L, Sawers Y, Loganathan R, Vacek JL, Stehno-Bittel L, Norikova L, Al-Jarrah M, Smirnova IV (2010). Electrocardiographic changes with the onset of diabetes and the impact of aerobic exercise training in the Zucker Diabetic Fatty (ZDF) rat. Cardiovasc Diabetol.

[B49] Wang G, Li W, Lu X, Bao P, Zhao X (2012). Luteolin ameliorates cardiac failure in type 1 diabetic cardiomyopathy. J Diabetes Complications.

[B50] Wang GG, Li W, Lu XH, Zhao X, Xu L (2013). Taurine attenuates oxidative stress and alleviates cardiac failure in type 1 diabetic rats. Croat Med J.

[B51] Wang K, Zhang L, Rao W, Su N, Hui H, Wang L, Peng G, Tu Y, Zhang S, Fei Z (2015). Neuroprotective effects of crocin against traumatic brain injury in mice: involvement of notch signaling pathway. Neurosci Lett.

[B52] Warne JP, Horneman HF, Wick EC, Bhargava A, Pecoraro NC, Ginsberg AB, Akana SE, Dallman MF (2005). Comparison of superior mesenteric versus jugular venous infusions of insulin in streptozotocin-diabetic rats on the choice of caloric intake, body weight and fat stores. Endocrinology.

[B53] Wayhs CA, Tortato C, Mescka CP, Pasquali MA, Schnorr CE, Nin MS, Barros HM, Moreira JC, Vargas CR (2013). The association effect of insulin and clonazepam on oxidative stress in liver of an experimental animal model of diabetes and depression. Pharm Biol.

[B54] Wu Z, Chen Q, Ke D, Li G, Deng W (2014). Emodin protects against diabetic cardiomyopathy by regulating the AKT/GSK-3β signaling pathway in the rat model. Molecules.

[B55] Zobali F, Avci A, Canbolat O, Karasu C (2002). Effects of vitamin A and insulin on the antioxidant state of diabetic rat heart: a comparison study with combination treatment. Cell Biochem Funct.

[B56] Zhang Z, Wang CZ, Wen XD, Shoyama Y, Yuan CS (2013). Role of saffron and its constituents on cancer chemoprevention. Pharm Biol.

[B57] Zheng H, Pu SY, Fan XF, Li XS, Zhang Y, Yuan J, Zhang YF, Yang JL (2015). Treatment with angiotensin-(1-9) alleviates the cardiomyopathy in streptozotocin-induced diabetic rats. Biochem Pharmacol.

